# Fetal Zone Steroids and Estrogen Show Sex Specific Effects on Oligodendrocyte Precursor Cells in Response to Oxidative Damage

**DOI:** 10.3390/ijms22126586

**Published:** 2021-06-19

**Authors:** Donna Elizabeth Sunny, Elke Hammer, Till Ittermann, Elisabeth Luise Krüger, Stephanie Hübner, Michaela Friederike Hartmann, Stefan Alexander Wudy, Uwe Völker, Matthias Heckmann

**Affiliations:** 1 Department of Neonatology and Pediatric Intensive Care, University of Medicine Greifswald, 17475 Greifswald, Germany; el.kruger@yahoo.de (E.L.K.); stephanie.huebner1@gmx.de (S.H.); matthias.heckmann@uni-greifswald.de (M.H.); 2 Department of Functional Genomics, University of Medicine Greifswald, 17475 Greifswald, Germany; hammer@uni-greifswald.de (E.H.); voelker@uni-greifswald.de (U.V.); 3 Institute for Community Medicine, University of Medicine Greifswald, 17475 Greifswald, Germany; till.ittermann@uni-greifswald.de; 4 Pediatric Endocrinology & Diabetology, Laboratory for Translational Hormone Analytics, Steroid Research & Mass Spectrometry Unit, Center of Child and Adolescent Medicine, Justus Liebig University, 35392 Giessen, Germany; michaela.hartmann@paediat.med.uni-giessen.de (M.F.H.); stefan.wudy@paediat.med.uni-giessen.de (S.A.W.)

**Keywords:** preterm birth, oligodendrocyte precursor cells, fetal zone steroids, estradiol, hyperoxia, sex-based difference, differentiation

## Abstract

Oxygen causes white matter damage in preterm infants and male sex is a major risk factor for poor neurological outcome, which speculates the role of steroid hormones in sex-based differences. Preterm birth is accompanied by a drop in 17β-estradiol (E2) and progesterone along with increased levels of fetal zone steroids (FZS). We performed a sex-based analysis on the FZS concentration differences in urine samples collected from preterm and term infants. We show that, in preterm urine samples, the total concentration of FZS, and in particular the 16α-OH-DHEA concentration, is significantly higher in ill female infants as compared to males. Since we previously identified Nup133 as a novel target protein affected by hyperoxia, here we studied the effect of FZS, allopregnanolone (Allo) and E2 on differentiation and Nup133 signaling using mouse-derived primary oligodendrocyte progenitor cells (OPCs). We show that the steroids could reverse the effect of hyperoxia-mediated downregulation of Nup133 in cultured male OPCs. The addition of FZS and E2 protected cells from oxidative stress. However, E2, in presence of 16α-OH-DHEA, showed a negative effect on male cells. These results assert the importance of sex-based differences and their potential implications in preterm stress response.

## 1. Introduction

Preterm birth is one of the leading causes of neonatal morbidity and mortality [1,2]. These babies are at a high risk of severe developmental disabilities as prematurity is associated with complex adverse consequences. Alterations in the pre-conceptional, fetal and early neonatal periods lead to profound phenotypic, endocrine and metabolic consequences. This includes perinatal exposure to oxidative stress which has been implicated in the pathology of a number of diseases related to prematurity, especially wide-spread white matter damage in the brain [3]. Moreover, there is accumulating evidence that females have an advantage over males with a better outcome in the perinatal period, particularly after preterm birth [4]. This gender-based difference points towards a possible role of hormones and, considering its relation to hyperoxia, it becomes important to identify the underlying reasons for differential responses.

During pregnancy, maternal progesterone level increases and the main source is the placenta. Apart from the placenta, the adrenal glands also synthesize progesterone under the control of adrenocorticotropic hormone (ACTH) due to which its secretion by the adrenal glands can increase in response to stress [5]. Not only progesterone, but 17β-estradiol (E2) plasma levels also increase up to 100-fold (15,000 pg/mL, approximately 55 nM) compared to the plasma estrogen concentrations in non-pregnant females [6,7]. The fetus is also exposed to increasing hormone levels during its development. The presence of high amounts of estrogen is considered to be critical for normal brain development [8,9]. Several experimental studies have highlighted that the female sex hormones represent potential neuroprotective agents against acute and chronic injuries in the brain [10,11,12]. But at the time of birth, after the umbilical cord is clamped, E2 level decreases by a factor of 100 within 24 h [13]. In humans, this estrogen synthesis by the placenta during pregnancy is largely dependent on androgens from the fetal and maternal adrenal glands. Fetal adrenal steroids are the source of about half of the estrone and estradiol and 90% of the estriol in the maternal circulation [14]. The human fetal adrenal cortex is an active endocrine organ that produces steroid hormones, which regulate intrauterine homeostasis and the maturation of the fetal organ system. An active steroidogenic specialized compartment of the fetal adrenal cortex is called the fetal zone, whose mass increases in utero up to term and then declines rapidly after birth. The fetal zone, together with the placenta, forms an effective steroid exchange channel and this functional association is termed as the “feto-placental unit” [15,16].

As a result of preterm birth, the feto-placental unit is disrupted and the synthesis of estrogen and progesterone in the placenta stops. However, the fetal zone of the preterm infant continues to synthesize steroid precursor molecules which are collectively called fetal zone steroids (FZS). FZS mainly comprise of dehydroepiandrosterone (DHEA), which is the main precursor molecule for estrogen synthesis. This DHEA is converted into more hydrophilic metabolites that are eventually excreted from the body through urine. We and others have reported that the urinary excretion of 3β-OH-5-ene steroids in preterm born infants is significantly higher for a longer period of time and does not approach that of term infants before 40 weeks postmenstrual age [17,18]. Additionally, a persistent higher plasma concentration of DHEA-sulfate (DHEAS) was found in preterm infants from the day of birth until 10 months of age, when compared to that of term infants [19]. Studies have reported that, even though high DHEAS levels during the first week of life may reflect the sudden discontinuation of the placental metabolism of circulating DHEAS into estrogens at birth, it might also partly represent a stress response [20]. Since preterm birth is accompanied by a drop in estrogen level and an increase in the levels of these precursor molecules to which the developing brain is normally exposed, the precise role of these precursor molecules in brain development needs to be addressed. If investigated in detail, these molecules might as well prove to be novel but endogenously synthesized neuroprotective agents that can promote cell survival and maturation.

By means of our previous study, we have experimentally shown that these FZS show a neuroprotective effect comparable to that of E2, in a hyperoxia induced cell death model of immature glial cells [21]. However, these experiments did not consider sex differences. Therefore, we have recently demonstrated that oxidative stress severely affects cellular functions related to energy metabolism, stress response, and maturation in male derived mouse primary oligodendrocyte progenitor cells (OPCs), whereas the female cells remain largely unaffected. We also uncovered Nup133 as a novel regulator and have shown how OPC differentiation and nuclear respiratory factor 1 (Nrf1) oxidative stress responses are mediated differentially by Nup133 and the classical estrogen receptor alpha (ERα) in male and female derived OPCs [22].

In the current study, we address the concentration changes in urinary FZS concentrations in preterm and term infants by classifying them based on gender and wellness. Further, we show the differential response of male and female derived OPCs to oxidative stress in presence of FZS, allopregnanolone (3α-Hydroxy-5α-pregnan-20-one; Allo) and E2. In the context of Nup133 signaling, we explain how the FZS, Allo and E2 have a neuroprotective role but at the same time, a differential effect on the maturation of male and female derived primary OPCs. Based on the results, we also project a possible explanation for the differences in urinary steroid profiles observed in preterm infants and their potential implications.

## 2. Results

### 2.1. Fetal Zone Steroid Amounts Differ in the Urine of Preterm Male and Female Infants

Our previous experimental data suggested endogenous neuroprotection by FZS [20] which are synthesized in high concentrations postnatally in preterm infants [17]. In humans, these steroid hormone precursors might play potential roles in brain development and in combating stress. Even though there have been studies about the neuroactive nature of some of these steroids in different models [23], their abundance in preterm infants has not yet been characterized completely. This includes characterization of the prevalence of these FZS in different body fluids and tissues based on gestational age, sex, illness, etc. We, therefore, analyzed urine samples collected from human preterm and term infants along a period of time starting from day 1 to day 3 after birth using GC–MS and characterized the data with respect to sex and illness.

Differences in fetal zone steroid median concentrations between well terms and well preterms were visualized by box plots. Associations between groups of infants (ill preterms, well preterms and terms) with corresponding fetal zone steroid concentrations were analyzed by median regression models in the whole population as well as stratified by sex.

**Figure 1:**
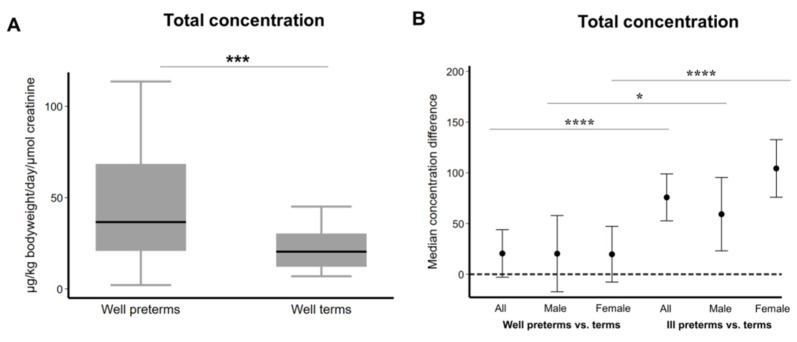
Fetal zone steroids (FZS) are excreted in high amounts in preterm infant urine. (**A**) Total FZS excretion in healthy term (>37 weeks gestational age, *n* = 42) and healthy preterm (<37 weeks gestational age, *n* = 71) infants during the first three days after birth measured in µg/kg bodyweight/day/µmol creatinine. *** *p* < 0.001 as determined by Wilcoxon–Mann–Whitney test. Median and 95% confidence interval are given. (**B**) A comparison of the total FZS excretion in healthy male preterm (<37 weeks gestational age, *n* = 42) and healthy female preterm (<37 weeks gestational age, *n* = 29) infants compared to sick male preterm (<37 weeks gestational age, *n* = 38) and sick female preterm (<37 weeks gestational age, *n* = 26) infants during the first three days after birth. * *p* < 0.05, **** *p* < 0.0001(median regression models). The beta coefficients and the belonging 95% confidence intervals were plotted. A whole 95% confidence interval above 0 is considered statistically significant.

The urinary steroid profile showed a significantly higher level of total FZS in the urine of well preterm infants when compared to well term infants (Figure 1A). Upon analyzing the concentration profiles in well and ill preterm infants stratified by sex, we found that there was no difference in the total FZS levels in well preterm male and female groups; whereas, when comparing between the well and ill groups, FZS levels were significantly higher in both ill male and female infants, with a still higher concentration (non-significant) in ill female infants as compared to males (Figure 1B).

### 2.2. Concentration of 16α-OH-DHEA and Other FZS Increases in Ill Female Preterm Infants

**Figure 2:**
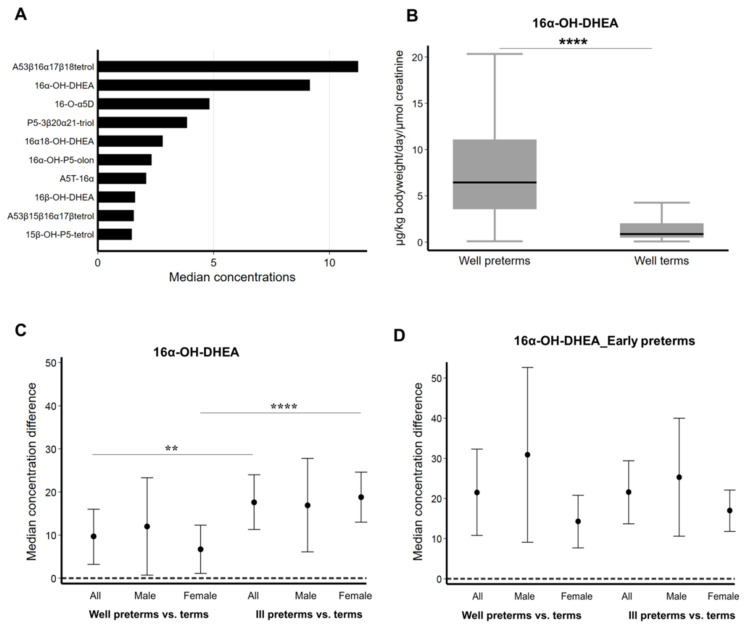
Urine concentration of selected FZS stratified by sex and wellness. (**A**) Concentrations of the ten highest analyzed FZSs during the first 3 days after birth in all preterm infants (well and ill, *n* = 135). (**B**) 16α-OH-DHEA concentration in preterm (*n* = 135) and term (*n* = 63) infants. **** *p* < 0.0001 as determined by Wilcoxon–Mann–Whitney test. Median and 95% confidence interval are given. (**C**) 16α-OH-DHEA excretion in well male preterm (<37 weeks gestational age, *n* = 42) and well female preterm (<37 weeks gestational age, *n* = 29) infants compared to ill male preterm (<37 weeks gestational age, *n* = 38) and ill female preterm (<37 weeks gestational age, *n* = 26) infants during the first three days after birth. ** *p* < 0.01, **** *p* < 0.0001 (median regression models). (**D**) 16α-OH-DHEA excretion in well male early preterm (<30 weeks gestational age, *n* = 7) and well female early preterm (<30 weeks gestational age, *n* = 10) infants compared to ill male early preterm (<30 weeks gestational age, *n* = 23) and ill female early preterm (<30 weeks gestational age, *n* = 21) infants during the first three days after birth. The beta coefficients and the belonging 95% confidence intervals were plotted. A whole 95% confidence interval above 0 is considered statistically significant.

**Figure A1:**
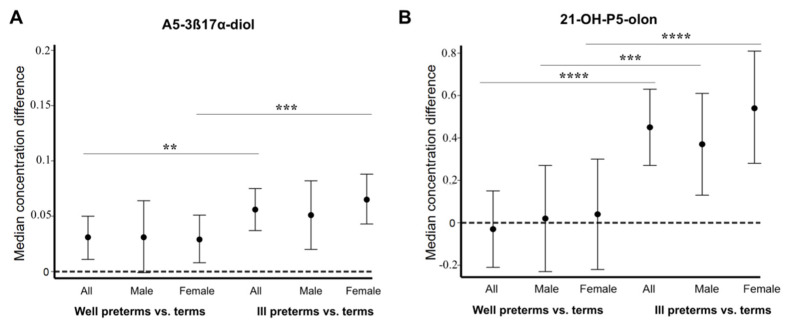
Concentration differences of selected individual FZS. Comparison of (**A**) A5-3β17α-diol and (**B**) 21-OH-P5-olon excretion in healthy male preterm (<37 weeks gestational age, *n* = 42) and healthy female preterm (<37 weeks gestational age, *n* = 29) infants compared to sick male preterm (<37 weeks gestational age, *n* = 38) and sick female preterm (<37 weeks gestational age, *n* = 26) infants during the first three days after birth. ** *p* < 0.01, *** *p* < 0.001, **** *p* < 0.0001 (median regression models). The beta coefficients and the belonging 95% confidence intervals were plotted. A whole 95% confidence interval above 0 is considered statistically significant.

As it is known, 16α-OH-DHEA is a precursor of 16α-hydroxylated estrogens. DHEA and DHEAS produced by the fetal zone are the most abundant circulating steroids; they are first hydroxylated at 16α-position in the fetal liver to form 16α-OH-DHEA and 16α-OH-DHEAS, and are finally aromatized in the placenta following transport across the feto-placental barrier [24,25]. In our data, 16α-OH-DHEA was found to be present in very high concentrations when compared to the other detected metabolites in the preterm group (Figure 2A), and significantly higher in the healthy preterm group than in the terms, (Figure 2B) with values going up to 17.5 (6.1–154.3) µg/kg bodyweight per day and µmol creatinine (*n* = 7) at 25 weeks gestational age compared to 0.6 (0.1–1.8) µg/kg bodyweight per day and µmol creatinine (*n* = 20) at 39 weeks gestational age. Further comparison based on sex and being well showed that 16α-OH-DHEA was present in significantly higher concentration in all ill preterm infants than the healthy ones. A particularly higher significant difference was observed between healthy and ill preterm females (Figure 2C). However, upon considering only the early preterm infants (<30 weeks gestational age), we saw that 16α-OH-DHEA was slightly higher in the healthy as well as sick male infants (Figure 2D). Additionally, we also looked at the concentration differences in the most abundant DHEA-metabolite androstene-3β, 17α-diol and the pregnenolone metabolite 21-OH-P5-olone. In the case of androstene-3β, 17α-diol we found a similar trend as 16α-OH-DHEA with significantly higher concentration in all ill preterm infants as well as in the case of ill female preterm infants when compared to all healthy preterm and the healthy female preterm group respectively (Appendix A Figure A1A). But in the case of 21-OH-P5-olone, although the concentrations in well preterm infants were seen to be almost similar to the term group, it showed significantly higher levels in male as well as female ill preterm infants when compared to their well counterparts (Appendix A Figure A1B). Hence, in an attempt to understand the consequences of these observed differences in FZS concentrations, we tested 16α-OH-DHEA and Adiol (androstene-3β, 17β-diol) as proxies of the FZS versus estradiol and allopregnanolone on the mouse OPCs.

### 2.3. FZS and Allopregnanolone Have a Protective Effect on Both Male and Female OPCs

**Figure 3:**
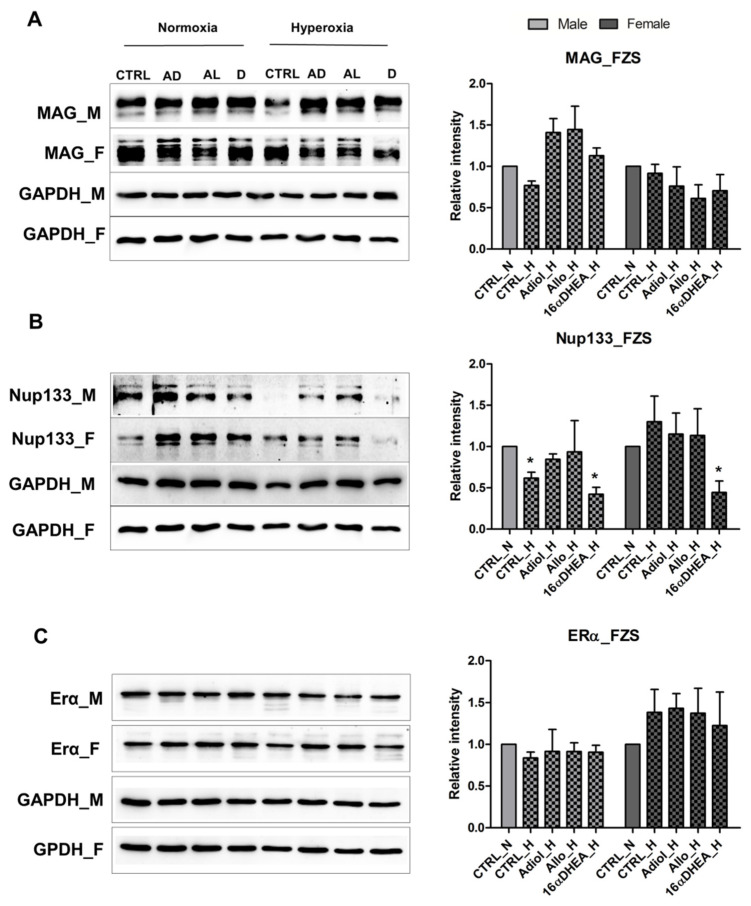
FZS and E2 show discrete effects on male and female OPCs. Western blot results showing the expression of (**A**) MAG, (**B**) Nup133 and (**C**) ERα in OPCs after treatment with 100 nM Adiol (AD), 100 nM Allopregnanolone (AL) and 100 nM 16α-OH-DHEA under normoxia and hyperoxia. All data are representative of at least three independent experiments. Bars and error represent mean ± SEM of replicate measurements. Significant differences from normoxia control are indicated by * *p* < 0.05 (Student’s *t*-test).

To evaluate the impact of hormones on the neurological development, we tested the effect of 16α-OH-DHEA along with Adiol (androstene-3β, 17β-diol) and Allo on the maturation of OPCs. As described previously, using a primary mouse-derived OPC culture model, we performed experiments with separate male and female derived OPCs simultaneously. The cells were subjected to an optimum treatment regime of 80% oxygen shock for 24 h and were then returned back to normal oxygen conditions and were allowed to recover and differentiate for 4 days. At the time of O_2_ shock, the OPCs were largely immature and vulnerable to oxygen damage. As we have previously shown, the male OPCs are severely affected by hyperoxia leading to impairment of maturation whereas the female cells remain largely unaffected [22]. This impairment of maturation was accompanied by the downregulation of the nucleoporin protein Nup133 and its direct downstream target nuclear respiratory factor 1 (Nrf1) in the male cells. The female cells seemed to be rendered resistant due to synergistic protection via the estrogen receptor alpha (ERα) which was upregulated following hyperoxia in female but not in male cells. Both Nup133 and ERα regulate mitochondrial function and oxidative stress response by transcriptional regulation of Nrf1 [22]. It was interesting to see that the administration of FZS and Allo had a positive effect on the maturation of male OPCs post hyperoxia (Figure 3A), as seen by changes in myelin associated glycoprotein (MAG) levels. In the case of female cells, considering the fact that the oxygen treatment itself did not show any reduction in MAG levels, treatment with the steroids seems to have broadly no additional effect. Similarly, under normoxic conditions, where protein levels after treatment with all three steroids were increased in the case of male cells, in the female derived cells they were mostly comparable to normal levels (CTRL_N). On looking at the expressions of Nup133 and ERα, we see that the ERα protein abundance tends to increase in the female cells under hyperoxic conditions when treated with the steroids, whereas in the male cells it remains unchanged. On the other hand, Nup133 expression shows a trend towards increase after treatment with Adiol and Allo in both male and female cells under hyperoxia, but not when cells were treated with 16α-OH-DHEA. This shows that the observed effect on maturation might be mediated not only by Nup133 but also by ERα in the female cells, as previously hypothesized [22]. Whereas, in male cells, this effect seems to be mediated mainly by Nup133. However, 16α-OH-DHEA seems to differ in its mechanism of action (Figure 3B,C). Hence, we show that these steroids have a positive effect on stress response and maturation post hyperoxia, especially in the male cells, and at the same time they can have discrete mechanisms of actions.

### 2.4. Estradiol in Presence of 16αOH-DHEA Exerts a Negative Effect on the Maturation of Male Derived Cells

**Figure 4:**
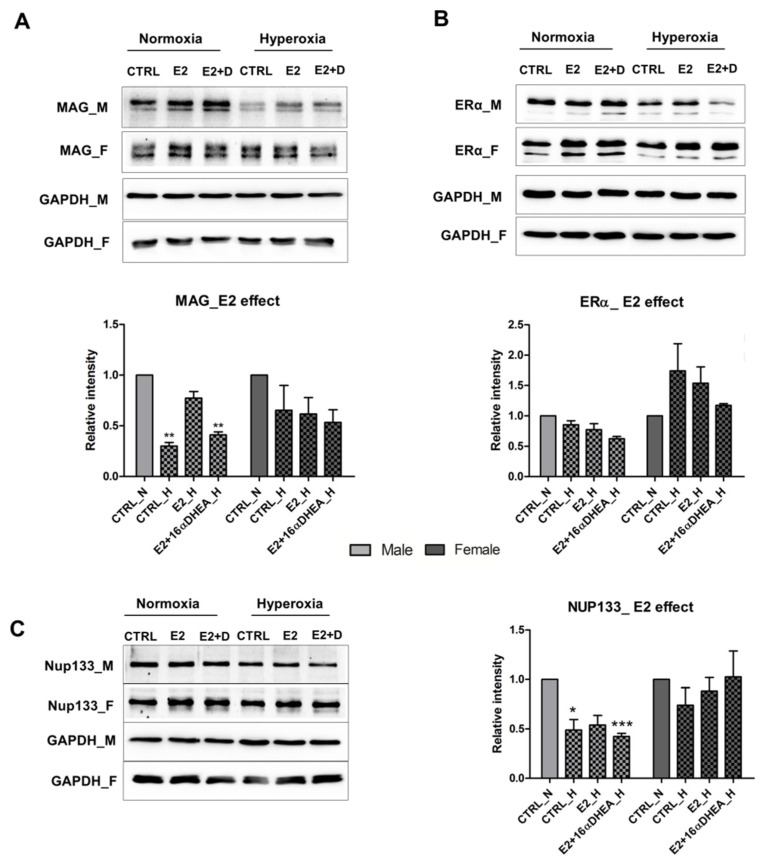
Effect of E2 and 16αOH-DHEA co-treatment. Western blot results showing the expression of (**A**) MAG, (**B**) ERα and (**C**) Nup133 in OPCs after treatment with 100 nM 17-β estradiol (E2) alone and E2 in combination with 100 nM 16α-OH-DHEA (E2+D) under normoxia and hyperoxia. All data are representative of at least three independent experiments. Bars and error represent mean ± SEM of replicate measurements. Significant differences from normoxia control are indicated by * *p* < 0.05, ** *p* < 0.01, *** *p* < 0.001 (Student’s *t*-test).

Since preterm birth is associated with a drop in estrogen and progesterone levels, it has been advocated that substituting estrogen to intrauterine levels can be beneficial [13,26,27,28]. These preliminary clinical studies to substitute these steroids were carried out in female preterm infants only and have failed to confirm significantly improved neurological outcomes. Considering the high amounts of circulating FZS in preterm infants, more experimental evidence is required to decipher the effect of any additional steroid substitution in the presence of FZS and its implications in male and female infants with respect to neuroprotection. In that context, we have previously shown that the FZS might provide endogenous neuroprotection which could interfere with E2-mediated protection [21]. However, we did not investigate any sex-based differences and therefore, here, we checked the effect of 16α-OH-DHEA on the differentiation of OPCs in the presence of E2 (co-treatment). We treated male and female OPCs with 100 nM E2 alone and in combination with 100 nM 16α-OH-DHEA. When giving high oxygen treatment for 24 h, we found that E2 had a positive effect on MAG expression in the male cells which was, however, diminished by co-treatment (Figure 4A) even though under normoxic conditions the co-treatment increased MAG expression. We checked the expression of Nup133 and ERα under the same treatment conditions and found that the abundance of both these proteins was decreased in the co-treatment group in male cells post hyperoxia (Figure 4B,C). Whereas in the female cells, an increased abundance was observed in the case of both Nup133 and ERα (Figure 4B,C). In general, increased protein levels were observed under normoxic conditions. This negative effect of the co-treatment on the male cells, along with the observed higher levels of 16α-OH-DHEA in the urine of early (<30 weeks gestational age) male preterm infants in both well and ill groups (Figure 2D), again points towards the importance of sex differences with respect to circulating FZS while considering hormone substitutions as a therapy in preterm infants. Table 1 summarizes the differences between co-treatment and single steroid treatments.

### 2.5. 16α-, OH-DHEA Activates Different Mechanisms in Male and Female Cells

**Figure 5:**
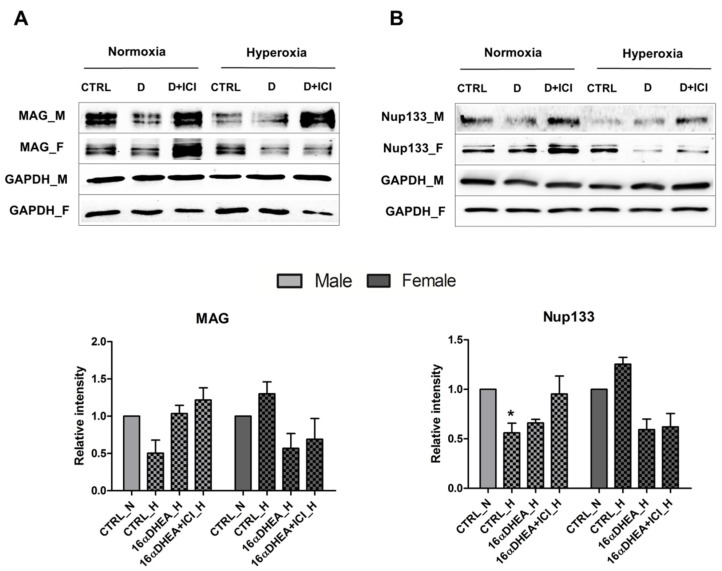
Mechanism of action of 16α-OH-DHEA in male and female OPCs. Western blot results showing the expression of (**A**) MAG and (**B**) Nup133 in OPCs after treatment with 100 nM 16α-OH-DHEA alone and 16α-OH-DHEA in combination with 1 µM ICI (D+ICI) under normoxia and hyperoxia. All other data are representative of at least three independent experiments. Bars and error represent mean ± SEM of replicate measurements. Significant differences from normoxia control are indicated by * *p* < 0.05 (Student’s *t*-test).

In our previous study we evaluated the receptors involved in the protective effect observed by FZS and found that the neuroprotection by FZSs was highly dependent on the cell type-specific expression of aromatases, the receptor repertoire and the potency of the fetal zone steroids towards these receptors [21]. However, in the primary OPCs we found an upregulation of ERα as a response to hyperoxic treatment in the female cells [22] and, therefore, to understand the sex specific effect of 16α-OH-DHEA on this ER response, we performed a co-treatment of 16α-OH-DHEA with ICI 182, 780 (ICI) which is an estrogen receptor antagonist. In the male derived cells, the co-treatment resulted in an increase in the protein levels of MAG as well as Nup133 under normoxic and hyperoxic conditions. Whereas, in the female cells, the increase observed under normoxic condition was diminished upon hyperoxia. (Figure 5). Treatment with ICI alone did not significantly impact MAG expression, except upon hyperoxic conditions in female cells, where a reduced MAG expression was observed. Hence the observed changes in the co-treatment seem to be attributed to 16α-OH-DHEA. This shows that 16α-OH-DHEA might additionally activate other steroid hormone receptors apart from ERα and ERβ. Hence, as observed throughout, it seems that 16α-OH-DHEA has profound differences in its mechanism of action and that it could possibly have different interactions in the male and female cells. Therefore, it would be intriguing to further explore the stress response signaling evoked by 16α-OH-DHEA which might eventually be critical for designing new neuroprotective measures.

## 3. Discussion

Following preterm birth, the brain is left extremely vulnerable as it is deprived of essential supplies from the placenta including steroids, enzymes and nutrients and at the same time is prematurely exposed to stimulating environments such as hyperoxia and associated damage [29]. Steroid hormones have been long implicated in neuroprotection and so are some of the major steroid hormone precursor molecules such as DHEA and the neuroactive steroid Allopregnanolone, which are being increasingly investigated for their pharmacological importance [23,30]. Even though DHEA is one of the major secretory products of the human adrenal gland, the concentration of DHEA has been found to be particularly high in the brain. It is the glial cells in the brain that either metabolize DHEA from blood born precursors or synthesize it *de novo* from cholesterol, independent of peripheral steroidogenic sources [31,32]. Unlike DHEA, Allo and its biosynthetic enzymes, 5α-reductase type I and 3α-hydroxysteroid dehydrogenase, are expressed in neurons but not in the glial cells of rodent and human brain [33]. However, reduced concentrations of Allo have been linked to reduced myelination [34]. In preterm infants, even though there are high circulating levels of steroid precursors [35], their role in neuroprotection is not well defined and neither are their possible differential effects on male and female subjects.

Previous works from Heckmann et al. and other working groups [35,36] have shown that fetal zonal steroids are synthesized in persistent high concentrations in premature babies for a considerable long duration. In this study, we performed a sex-based analysis on the concentration differences of FZS in preterm and term infants, and also in ill and well preterm infants, using the data collected from the first 3 days after birth. This period immediately after birth is characterized by the highest illness severity in preterm infants and therefore, this critical time period was specifically included for analysis in order to obtain a clearer picture of the differences in steroid concentrations that would be more relevant to be considered while making any correlations with experimental results. The data revealed striking differences between the male and female groups. The ill female infants showed a significant higher amount of total FZS. Previous studies on animal models have very clearly shown that preterm born male animals had reduced myelination and showed immaturity of the oligodendrocyte cell lineage after birth. The observed neurodevelopmental delay remained in the preterm male offspring even at term equivalent age [37]. Our data points towards the possibility that in the case of ill preterm infants, especially female infants, due to yet unknown reasons, the production of FZS by the adrenal cortex is still much more active than their healthy counterparts. And if this higher amount has been found in the urine, it might be very well possible that there exists a corresponding higher level in circulation as well. Therefore, this could lead to the buildup of a transient endogenous neuroprotection, rendering the female infants more protected towards the damages caused by perinatal stress. However, the role of individual steroids still need to be investigated in detail in both the sexes separately to understand the complex mechanisms behind any overall neuroprotective effect.

We have previously observed neuroprotective effects by estradiol, DHEA, 16αOH-DHEA and Adiol in a model of hyperoxia-mediated cell death in the female derived immature oligodendroglial cell line (OLN93) [21]. We saw that DHEA, 16αOH-DHEA and Adiol activated ER-α and/or ER-β as a result of which a significant protection was achieved. At the same time, a co-treatment with estradiol in the presence of FZS did not add up to the already observed protection by FZS alone. However, the exact mechanisms and sex-based differences were not investigated. Therefore, in order to find more about the mechanisms of hyperoxia induced injury in oligodendrocytes, we performed a sex-based study on the differences in OPC maturation post hyperoxia and the results have shown how differentiation and Nrf1-oxidative stress responses are mediated by Nup133 in the male cells and the involvement of the classical estrogen receptor alpha (ERα) as a possible upstream regulator of Nup133 [22]. We saw that there exists a difference in the extent of regulation via ERα in the male and female cells with a possible stronger regulation in the female cells allowing them to better cope with the oxidative stress. In this study, we checked the effects of FZS, Allo and E2 on this mechanism and the maturation of OPCs. We show that these steroids have a differential effect on the male and female cells. Specifically, in post hyperoxic treatment we see that the FZS and Allo showed a positive effect on the maturation of the male OPCs, whereas, in the female OPCs, considering that the hyperoxic treatment itself did not hinder their maturation, no additional effect was observed. This was consistent upon treatment with E2 and co-treatment with 16αOH-DHEA in the female cells, but a negative effect of co-treatment was observed in the male cells post hyperoxia. From the mechanistic point of view, post hyperoxia, an overall additional effect was observed in the female cells via ERα upregulation. These results also illustrate that the FZS could be potential ligands of ERα and might protect the OPCs against oxidative stress via ERα activation. However, the effect of 16αOH-DHEA was notable as it showed a positive effect on maturation post hyperoxia in the male cells, but at the same time showed a decrease in Nup133 abundance and no up-regulation of ERα (Table 1). Considering that the total excreted FZS concentration associates with circulating levels and are higher in ill preterm female infants than in ill male infants (Figure 1B), we propose that the presence of these FZS render the female cells more resistant to oxidative stress compared to the males. This also makes it worth investigating in detail any changes in the concentration of these FZS in male and female infants under specific disease conditions. An overall combination effect, as mentioned above, must also be considered. Especially, considering the high amounts of circulating 16αOH-DHEA in preterm infants, it is important to perform further experiments to decipher its mechanism towards stress response.

We assume that this could be linked to the sex-based difference in the neurological outcome that has been observed over various clinical studies [4,38,39,40]. The experimental results with FZS treatment performed on male and female cells separately supports this hypothesis and show that this differential effect should be considered crucial for designing clinically effective neuroprotective strategies. As our results show that these FZSs can activate different mechanisms in male and female cells, more detailed studies would still be required to uncover the role of these FZS as potential pleotropic molecules that can interact with a number of steroid hormone receptors activating a myriad of distinct or overlapping signaling and mechanisms.

This study also throws light on the possible consequences of substituting estrogen in preterm infants without considering the presence of high amounts of circulating FZS and that these consequences can be largely influenced by sex. Particularly, the experimental results very clearly show a negative impact of co-treatment with 16αOH-DHEA on the described mechanism in the male cells. Since all these steroid molecules can have individual discrete effects as well as completely different regulatory mechanisms when present together with respect to stress response, it is logical to study these combinatory effects of endogenous and supplemental steroid metabolites in details. Even though, in a human system the situation is much more complex, these results point towards the first clues that demand further studies using various model systems to understand these complex interactions and their subsequent consequences without ignoring the sex-based differential aspects. Any future studies aiming at steroid supplementations or steroid-based therapies in preterm infants should also study the effects of the proposed steroids in combination with FZS and especially 16αOH-DHEA.

## 4. Materials and Methods

### 4.1. Human Infant Urine Study

This study was enrolled at the Justus Liebig University, Giessen. The study population consisted of two cohorts of preterm infants (gestational age < 30 and 30–36 weeks) enrolled originally in a prospective longitudinally study to investigate adrenal glucocorticoid production by GC–MS in relation to illness and postnatal growth. [41,42]. Term infants with a gestational age >37 weeks were enrolled separately from March 2009 until July 2010.

Here, 24 h urine collections from the first three days after birth were analyzed as the severity of illness was expected to be highest during this period.

The urine collection procedure was performed as described previously [43]. In short, urine was collected for 24 h using disposable nappies composed of pure cellulose (Pampers; Procter & Gamble, Schwalbach, Germany). Urine was extracted by compressing the nappies using a hydraulic press applying a maximum of 120 kPa/cm^2^. After centrifugation, the collected urinary specimens were stored at −80 °C until analysis by GC–MS.

Urinary steroid profiles were determined by GC–MS analysis according to the procedure previously described [18]. A total of 17 FZS were determined by selected ion monitoring as listed in Table 2. To assess the overall secretion, these 17 major urinary metabolites were quantified (peak-area integration) and summed. Daily urinary excretion rates of the metabolites were corrected for body weight per micromoles creatinine to take into account changes in glomerular filtration rate during the first days of life [42].

Preterm infants with a gestational age of less than 30 weeks [17] and more than 30 weeks [23], who had no family history of adrenal illnesses and who had no major congenital anomalies, were eligible for the study. Postnatal steroid therapy was considered as a criterion for exclusion. Gestational age was determined using the expanded Ballard score and/or obstetrical dating.

Preterm infants were classified as being well according to previously described criteria [1]. Well infants had no signs of infection and did not receive treatment with surfactant or inotropes. Ill preterm infants suffered from one or more of the following diseases: respiratory-distress syndrome treated with surfactant, infection at birth (C-reactive protein >10 mg/L and symptoms of an infection during the first 72 h of life), hospital infection (C-reactive protein >10 mg/L and symptoms of an infection after the first 72 h of life), ventricular hemorrhage more than II° according to the criteria described by Papile et al. [2] (Papile et al., 1978). Term infants were classified as being well when they received only routine care after delivery in the well-baby nursery of the department of obstetrics and were not admitted to the department of neonatology in the children’s hospital.

### 4.2. Animals

Mouse protocols were approved by the Veterinary and Food Control Office, State Department of Agriculture, Food Safety and Fisheries Mecklenburg-Vorpommern, Germany (Permit Number: ZSF3936/11/17; Ethic approval date: 07 November 2017). Animal colonies were housed and maintained following the international FELASA, national GVsolas and local University Medicine Greifswald animal research guidelines. Crl:CD-1 (ICR) mice were received from the Charles River Company and bred for a maximum of two generations at the facility to obtain the donor mice and were used to generate oligodendrocyte precursor cells.

### 4.3. Isolation and Culture of Mouse OPCs

Mouse OPCs were isolated from enzymatically dissociated P2–P4 (postnatal day 2–4) old CD-1 (wild type strain) mice brains. For each isolation, tissue from the mid brain region comprising the sub ventricular zone from 3 male and 3 female (from the same litter) brains were pooled separately. The sex of the pups was determined visually and with genotyping. All animal usage for cell isolation was performed according to the institutional regulation regarding animal ethics.

Isolated cells were cultured in serum free growth medium supplemented with B-27(*w/o* retinol), EGF 0.02 µg/mL and FGF 0.005 µg/mL under 3% O_2_ conditions. After ~5–6 days when the neurospheres were around 100µm in diameter, the neurosphere growth medium was gradually replaced by serum free B104CM containing oligosphere medium every alternate day for 2 weeks. After this time period, the cells were trypsinized and used for experiments. Detailed protocol for the isolation and culture of mouse OPCs has been published in Nature Protocol Exchange [44].

### 4.4. Treatment with Hyperoxia

For differentiation experiments, the male and female derived OPCs were trypsinized separately, the cells were counted and seeded in equal densities into 6 well plates for adherent cells (without any coating; Cell+, Sarstedt, Nümbrecht Germany) containing 1 mL oligosphere medium per well. The cells were first given 80% O_2_ shock for 24 h, after which, 1 mL fresh media was added to each well and all plates were returned to 3% O_2_ (normoxia) condition. The cells were then allowed to differentiate for 4 days with 1 mL medium change every alternate day.

For protein studies, the cells from male and female derived OPCs were trypsinized, counted, and seeded in equal densities into 6 well plates for adherent cells (without any coating; Cell+, Sarstedt, Nümbrecht Germany) containing 1 mL DMEM/F12 + 45% glucose solution (666 µL for 50 mL DMEM/F12) medium per well. The plates were then kept at 80% O_2_, 5% CO_2_ and 37 °C (hyperoxia) and 3% O_2_, 5% CO_2_ and 37 °C (normoxia) incubators respectively, for 24 h. Cells were subjected to protein and RNA extraction immediately after the treatment time. All cells (adhered and suspended) from each well were used for further processing and analysis.

### 4.5. Treatment with Steroids

For differentiation experiments as well as for protein studies, the procedure was followed exactly as mentioned above with addition of FZS or E2 before giving the oxygen shock.

A final concentration of 100 nM was used for treatment in the case of all the steroid molecules tested.

The estrogen receptor antagonist, ICI 182, 780 (ICI) was administered in a final concentration of 1 µM.

### 4.6. Cell Lines

B104 Neuroblastoma cell line was purchased from the American Type Culture Collection (ATCC). The cells were used only for preparing conditioned media for OPC culture as described previously [44].

### 4.7. Immunoblot Analysis

Protein extracts from whole cell lysates containing ∼40 µg protein was loaded in each lane of a Mini-Gel module for electrophoresis (BioRad, Munich, Germany). Protein was transferred onto nitrocellulose membrane (Amersham Protran 0.45 µm NC Western Blotting Membrane, GE Healthcare, Chicago, IL, USA), blocked with 1× Blocking buffer (Pierce™ Protein-Free (TBS) Blocking Buffer, Thermo Fisher, Waltham, MA, USA) at RT for 1 h, and incubated in primary antibody at 4 °C overnight. GAPDH (Rabbit Anti-GAPDH (D16H11) mAb, Cell Signaling Technology, Danvers, MA, USA) was used as the loading control at 1:1000 dilution. The other primary antibodies used were: Mouse Anti-Estrogen receptor alpha (33) mAb (MA1-310), Thermo Fischer Scientific, Waltham, MA, USA; Rabbit Anti-MAG mAb (D4G3) (9043), Cell Signaling Technology, Danvers, Massachusetts, USA; Anti-Rabbit NUP133 Polyclonal(12405-1-AP), Proteintech, Rosemont, Illinois, USA. Blots were incubated with secondary antibody at 1:10,000 dilution in blocking buffer for 1 h at RT. Protein bands were visualized with SuperSignal™ West Femto Maximum Sensitivity Chemiluminescence Substrate (Thermo Fischer Scientific, Waltham, MA, USA). Densitometric intensities were calculated using ImageLab software (BioRad, Munich, Germany). GAPDH signal intensity was used for normalization of data before relative intensity to CTRL_N for each individual condition was calculated.

### 4.8. Experimental Design and Statistical Analysis

The statistical analysis for Western blot data was performed using GraphPad Prism 5 for Windows (Source: GraphPad Software, San Diego, CA, USA; http://www.graphpad.com/; accessed date: 14 April 2021 (RRID:SCR_002798). Data from all bio-replicates (minimum 3 for each experiment) for each experiment was plotted with the mean value and standard error bars (standard error of mean, SEM). For statistical comparison between two variables, unpaired Student’s *t*-test was used and One way ANOVA (one-way analysis of variance) with Tukey’s/Dunnet’s post-hoc test was applied to compare between multiple treatment groups. A variance with a *p* value < 0.05 was considered significant. Various levels of significance were depicted as follows: * *p* < 0.05; ** *p* < 0.01; *** *p* <0.001.

Details of the statistical analysis used for generating the urine steroid profile data are mentioned in the results section. Twenty-four-hour urinary steroid excretion rates of the first three days of life were summed to generate an integral parameter of FZS production. All analyses were performed using the Stata version 15.1 software (Stata Corporation, College Station, Texas, USA).

## Figures and Tables

**Table 1:** Impact of steroids on OPCs in comparison to the untreated normoxic control cells. For assessing the effects, the normoxia control has been considered as the base and the effects of the steroids on the male and female derived cells under hyperoxic conditions have been interpreted accordingly.

	Normoxia					Hyperoxia				
	Adiol	Allo	16αD	E2	E2 + 16αD	Adiol	Allo	16αD	E2	E2 + 16αD
**Impact on maturation**										
Male	**++**	**+++**	**+++**	**++**	**+++**	**++**	**+**	**+**	**no**	**–**
Female	**+**	**+**	**++**	**+**	**+**	**no**	**no**	**no**	**no**	**no**
**Impact on Nup133**										
Male	**+**	**+**	**+**	**+**	**no**	**+**	**+**	**–**	**no**	**–**
Female	**++**	**++**	**+**	**+**	**+**	**+**	**+**	**–**	**+**	**+**
**Impact on ERα**										
Male	**no**	**no**	**no**	**no**	**+**	**no**	**no**	**no**	**no**	**–**
Female	**+**	**+**	**+**	**++**	**++**	**++**	**++**	**+**	**++**	**++**

+/–—positive or negative effect, no—no effect, 16αD (16α-OH-DHEA).

**Table 2:** List of all 3β-hydroxy-5-ene-steroids identified in human infant urine samples.

	Metabolites	
1	Adiol	5-androstene-3β,17β diol
2	A5-3β17α	5-androstene-3β,17α diol
3	DHEA	5-androstene-3 βol-17-one
4	16α-OH-DHEA	5-androstene-3 β,16α-diol-17-one
5	16β-OH-DHEA	5-androstene-3β,16β-diol-17-one
6	15β16α-OH-DHEA	5-androstene-3β, 15β, 16α-triol-17-one
7	16-O-A5D	5-androstene-3β, 17β-diol-16-one
8	A5T-16α	5-androstene-3β,16α,17β-triol
9	A5T-16β	5-androstene-3β,16β,17β-triol
10	16α18-OH-DHEA	5-androstene-3β, 16α, 18-triol-17-one
11	15β17α-OH-P5-olon	5-pregnene-3β,15β,17α-triol-20-one
12	16α-OH-P5-olon	5-pregnene-3β,16α-diol-20-one
13	A53β16α17β18-tetrol	5-androstene-3β,16α,17β,18-tetrole
14	A53β15β16α17β-tetrol	5-androstene-3β,15β,16α,17β-tetrole
15	15β-OH-P5-tetrol	5-pregnene-3 β, 15β, 17α,20α-tetrol
16	21-OH-P5-olon	5-pregnene-3β,21-diol-20-one
17	P5-3β20α21-triol	5-pregnene-3β,20α,21-triol
18	P5-tetrol	5-pregnene-3β,16α,20α,21-tetrole
